# Current research on child maltreatment epidemiology

**DOI:** 10.1186/s13034-018-0228-1

**Published:** 2018-04-27

**Authors:** Andreas Jud

**Affiliations:** 1 Department of Child and Adolescent Psychiatry/Psychotherapy, University of Ulm, Steinhövelstr. 1, 89075 Ulm, Germany; 2 School of Social Work, Lucerne University of Applied, Sciences and Arts, Lucerne, Switzerland

## Editorial

Child protection research in general should inform parents and other caregivers on how to best provide a violence-free upbringing for their children. It should furthermore inform professionals on how to best protect and support victimized children and their families. Research on child maltreatment epidemiology has yet another, specific goal: it should inform policy-makers and administrators on how to best manage child protection systems, on how to improve early detection of child maltreatment and accessibility for high-risk groups, particularize and scale-up prevention programs, etc. Unfortunately, research on child maltreatment epidemiology still has many gaps: population surveys have so far primarily focused on the prevalence of child sexual abuse and only a handful of countries can build their child protection strategies and policies on evidence of nationally representative data of reported child maltreatment incidents. This special issue aims at contributing to bridge the gap on lacking child maltreatment epidemiological research. It provides an overview on current studies in this area, both on the prevalence of child maltreatment and reported incidents:

Witt et al. [1] report the most recent findings on the prevalence of child maltreatment in Germany. Surveying a representative sample of 2510 participants between the ages of 14 and 94 years in 2016, they corroborate previous studies in highlighting that having experienced an incident of child maltreatment, particularly neglect, is still rather common for residents in Germany: over 10% of participants reported at least moderate emotional neglect and even more than 20% at least moderate physical neglect. The decline of this most prevalent form for younger ages is, however, promising. For other forms of child maltreatment, the prevalence has not decreased with younger age of the participants. Many efforts are still needed to tackle child maltreatment in this high-income country.

In an unprecedented effort, Nikolaidis et al. [2] not only collected data on child maltreatment prevalence for a single country, but for a majority of countries in the Balkan region—Albania, Bosnia and Herzegovina, Bulgaria, Croatia, the Former Yugoslavian Republic of Macedonia, Greece, Romania, Serbia, and Turkey. The shared methodology of the Balkan Epidemiological Study on Child Abuse and Neglect (BECAN) allows for a reliable comparison of findings in these different countries. A total of 42,194 children at the ages of 11, 13 and 16 years participated and self-reported high rates of lifetime and past-year prevalence of child maltreatment. For all countries, beyond 50% of the sample reported a lifetime prevalence for both experiencing psychological and physical violence. In contrast to the majority of literature on child sexual abuse, several countries have higher rates of male sexual abuse compared to females.

Canadian researchers had a pioneering role in collecting incidence data on reported child maltreatment. For two decades, the Ontario Incidence Study on Reported Child Abuse and Neglect (OIS) surveyed a representative sample of child maltreatment investigations every 5 years. This exceptional source of child maltreatment incidence data is one of the few worldwide that is able to identify trends in child protection practice. The findings of Fallon et al. [3] show a substantial decrease in rates of reported child sexual abuse across waves which may indicate a “real” decline. Policy changes had an obvious impact on incidence rates. By introducing the new category “risk of future maltreatment”, reported incidents have almost doubled. If this approach helps to identify and support more families before violence happens and offer them the necessary support to address their problems, we should ultimately be able to reduce prevalence of child maltreatment.

Finally, Jud et al. [4] present lessons learned from the first nationwide study on agency response to all forms of child maltreatment in Switzerland. To reach a remarkably high participation rate of 76% of the contacted agencies, the researchers intensely collaborated with child protection practitioners: they listened to their input in designing the survey, worked together in creating a set of variables, and continued to exchange both formally and informally during the survey and beyond. Researchers who are planning a representative study on reported child maltreatment will likely be able to transfer some of these “good practice” examples to their own context.

Taken together, findings of these studies might help to trigger and inspire urgently needed future research on child maltreatment epidemiology. Furthermore, establishing university institutes and positions focusing on child protection research will also advance the field. At the University of Ulm, Germany, the state of Baden-Württemberg provides both resources for the Competence Center Child Abuse and Neglect and the first chair in German-speaking Europe on Child Maltreatment Epidemiology and Trends in Child Protection. I feel honored to have been selected for this position. Hopefully, other universities will follow and formally implement child protection research as a part of their portfolio—a field of research that has the potential to contribute to children’s lives free of violence.

## References

[1] Witt A, Brown RC, Plener PL, Brähler E, Fegert JM (2017). Child maltreatment in Germany: prevalence rates in the general population. Child Adolesc Psychiatry Ment Health..

[2] Nikolaidis G, Petroulaki K, Zarokosta F, Tsirigoti A, Hazizaj A, Cenko E (2018). Lifetime and past-year prevalence of children’s exposure to violence in 9 Balkan countries: the BECAN study. Child Adolesc Psychiatry Ment Health..

[3] Fallon B, Trocmé N, Filippelli J, Black T, Joh-Carnella N (2017). Responding to safety concerns and chronic needs: trends over time. Child Adolesc Psychiatry Ment Health..

[4] Jud A, Kosirnik C, Mitrovic T, Ben Salah H, Fux E, Koehler J (2018). Mobilizing agencies for incidence surveys on child maltreatment: successful participation in Switzerland and lessons learned. Child Adolesc Psychiatry Ment Health..

